# Editorial: Mental State Understanding: Individual Differences in Typical and Atypical Development

**DOI:** 10.3389/fpsyg.2017.01183

**Published:** 2017-07-13

**Authors:** Daniela Bulgarelli, Anne Henning, Paola Molina

**Affiliations:** ^1^Dipartimento di Psicologia, Università degli Studi di Torino Turin, Italy; ^2^SRH Hochschule für Gesundheit Gera Gera, Germany

**Keywords:** theory of mind, emotion understanding, social cognition, mentalization, typical development, atypical development

We often refer to mental states such as intentions, desires, and beliefs to explain and predict our own behavior and that of others. Mental state understanding develops from infancy through adolescence and adulthood. A deeper understanding of influencing developmental factors may be obtained by studying individual differences in typical and atypical populations.

The current Research Topic addresses several topics about mental state understanding and development in childhood. It is organized into three sections, comprising 18 papers in total.

The first section addresses the development of social cognition in typical populations through seven papers. Different from most research on Theory of Mind (ToM) that commonly focuses on age-related changes, Blijd-Hoogewys and van Geert investigated whether there occur non-linearities during ToM development in childhood. Within an overall developmental trend that leveled off toward the age of 10 years, results showed two non-linearities suggesting a developmental shift in ToM understanding: a stagnation at the age of around 4 years and 8 months and a dip at the age of 6 years to six and a half years.

Four papers concern influencing social factors on children's ToM. Rosso and Airaldi showed that maternal reflective functioning (but not maternal attachment security) predicted their preadolescent child's reflective functioning, and that maternal ability to metalize mixed-ambivalent mental states predicted the corresponding ability in their child. While maternal education and linguistic competence are well researched influencing factors (e.g., NICHD HLB, 1998; Pons et al., 2003; Sammons et al., 2004), Bulgarelli and Molina showed that preschooler's linguistic competence mediated the effect of maternal education. Moreover, center-base care in the first 3 years of life eliminated the effect of maternal education, suggesting a protective role of center-base care for children with less educated mothers. Göbel et al. assessed the relation between emotion understanding and internalizing and externalizing behavior in 7- to 10-year-old children in a non-clinical, community sample. Inconsistent with prior research, the overall level of emotion understanding, comprising nine components, was not related to externalizing symptoms, but correlated positively with elevated levels of somatic complaints and anxious/depressed symptoms. Also, and specifically, higher levels of social withdrawal were associated with worse performance in understanding emotions elicited by reminders. Pinto et al. showed that joint narratives only improve 6- to 10-year-old's children's mental state talk performances when children were at the moment of initial elaboration or emergence of mental state talk, and when intersubjectivity levels were high, that is, when children produced more utterances to orchestrate and regulate the dialog.

Other two papers concern possible implications of ToM development on social interaction. Bosco and Gabbatore suggested that first-order ToM may play a causal role in explaining 3- to 8-year-old children's performance in handling pragmatic phenomena, namely sincere and deceitful speech acts. As to children's cognitive performances in social interaction regarding spatial tasks, Viana et al. showed that 5- to 9-year-old children's ToM was a better predictor of their spatial performances in a dyadic condition than their age, gender, and spatial performances in an individual setting.

Overall, the papers regarding typically developing children present some interesting ideas about the development of understanding mental states. This competence proves to be linked with different aspects of development, at social, cognitive, and relational levels: Rosso and Airaldi showed that only maternal reflective functioning, and not maternal attachment security, predicted children's mental state understanding; in turn, the paper of Bulgarelli and Molina confirmed the role of language and that of Pinto et al. the role of communicative context on children's ToM. Understanding mental states shows to be a complex ability that involves different functions and effects different aspects of development. Finally, the contribution of Blijd-Hoogewys and van Geert presented an interesting new approach to the study of ToM development that has implications for the debate whether this development may be stage-like or continuous.

The second section of this Research Topic encompasses nine papers that address the development of mental states understanding and its correlates in atypical populations. The possibility to compare results derived from studies carries out with typical and atypical populations is of key importance. In fact, similarities and differences in typical and atypical development can shed light on the processes at the base of the ability to understand, attribute and interpret mental states.

Lábadi and Beke's study concerned the role of structural connectivity across the hemispheres in neurodevelopmental disorders. They showed that 6- to 8-year-old children with agenesis of the corpus callosum exhibited mild impairments in recognizing emotions and in understanding theory of mind, and also showed more behavioral problems than control children matched by IQ and sociodemographic variables.

White et al. showed differential effects of social exclusion on children's usage of their capacity to understand mental states in relation to anxiety. After children were non-accidentally excluded in a virtual game, typically developing 5-year-olds' (Study 1) completion of peer-scenario stories were characterized by portraying story-characters more strongly as intentional agents, with use of more mental state language, and more between-character affiliation. Differently, 4- to 8-year-old children with anxiety disorder (Study 2) told stories in which story-characters exhibited less intentionality and less use of mental-state language. Thus, while exclusion may induce young children to mentalize, and thus to more effectively reconnect with others, excessive anxiety may impair this usage of controlled mentalizing.

The study by Amadó et al. investigated the relation between social cognition and executive functioning in children with Down Syndrome (DS). Children with DS were delayed in social cognition and in executive functioning, with unequal impairment of different functions. Moreover, working memory explained a higher amount of variability in social cognition performance than in typically developing children matched by age.

Implicit mentalizing consists of a spontaneous anticipation of an agent's false belief-based action that can be observed through anticipatory looking biases in tasks where eye movements are assessed. Using eye tracking devices, Schuwerk et al. showed that implicit mentalizing persists over infancy up to childhood in typical population; on the contrary, children with Autistic Spectrum Disorder (ASD) appeared to be impaired in such skill, even when their performance in the explicit tasks were similar to the matched control group. The results of this study–intact explicit mentalizing, impaired implicit mentalizing and no relation between that and executive function in children with ASD−support theories that propose two dissociable mentalizing systems.

The review by Margoni and Surian and its corrigendum discussed the idea that impairment in mental state understanding is the main factor explaining why children with ASD face difficulties in moral judgements: in fact, these children mainly rely on actions consequences and other external factors rather than on the agents' mental states when solving moral reasoning tasks.

Due to restricted discussion of abstract concepts, and to a possible to mismatch between language capabilities of children and their parents, the literature reported that deaf and hard-of-hearing signing children can display delays in mental states development (Peterson, 2009). Wang et al. compared children with a cochlear implant or a hearing aid with normally hearing participants matched by age and gender and showed that children with cochlear implants and hearing aids were developmentally delayed not only in verbally labeling the facial expressions of happiness, sadness, anger, and fear, but also in a non-verbal emotion-matching task. Holmer et al. showed that deaf and hard-of-hearing signing children were delayed in ToM tasks performances; only three of them have been exposed to sign language since birth. ToM was associated with reading comprehension and working memory, but not with sign language comprehension.

The inter-relation between language and ToM has been clarified in a meta-analysis by Milligan et al. (2007). Deepening this relation in children with Specific Language Impairment (SLI) is interesting, because some studies found delays in this population while others did not (Perner et al., 1989; Shields et al., 1996; Bulgarelli and Molina, 2013). In the review by Vissers and Koolen preschoolers with SLI appeared to be impaired both in cognitive ToM (imitation, joint attention, false belief understanding) and in affective ToM (recognizing and understanding emotions).

The review by Zmyj et al. addressed the role of joint attention as a precursor of social cognition, focusing on pre-term born children: they were less likely to initiate joint attention with others and to respond to others' attempts of engagement. The authors suggest that these deficits in joint attention might lead to impairments in social cognition, and in social interaction skills.

Deficits in mental states understanding are reported for children with different developmental disorders or impairments, from neurological ones (agenesis of the corpus callosum), prematurity, ASD, and personality difficulties such as anxiety. The paper of Schuwerk et al. suggested an interesting topic for future research: the possibility to differentiate implicit from explicit ToM based on different results in typical and ASD populations. On the contrary, as in typical development, the role of language is supported also by the present studies on children with SLI (Vissers and Koolen) and hearing impairment (Holmer et al.; Wang et al.).

A third and final section in this Research Topic is composed by two papers regarding evaluation and training tools. Valle et al. presented the “Thoughts in Mind (TiM) Project” that aimes at training mentalizing skills in adults (e.g., teachers and parents) to positively affect children's mentalization. They reported first evidence of the efficacy of the training when done with teachers: only the TiM Project training group significantly improved in third order false belief understanding and in two of the three components of a Mentalizing Task. Herbort et al. presented a new tool to assess ToM, the ToMenovela, that consists of 190 scenes depicting daily-life situations, addressing cognitive and affective ToM, emotional reactivity, and complex emotion judgment with respect to Ekman's basic emotions. First results on the use of the test with neurologically and psychiatrically healthy adults were reported. The tool proposed by Herbort et al. is very interesting because tools assessing adults' ToM are very scarce. Valle et al. proposed a teacher's training effective in improving children's abilities: a relevant aspect of research in mental states understanding effectiveness.

The current Research Topic addressed the development of mental state understanding in children with typical and atypical population, and reported new suggestions about the way to evaluate it and to support it through training. The presented frame was multifaceted. In respect to typical populations, the role of maternal reflective functioning, language, communication, and educational contexts has been deepened; and the association with internalizing/externalizing behaviors, performances in spatial tasks and pragmatics has been addressed as well. As to atypical populations, deficits in mental states understanding were reported for children with different developmental disorders or impairments, as the agenesis of the corpus callosum, Down Syndrome, prematurity, ASD, hearing impairment and personality difficulties such as anxiety.

## Author contributions

DB, AH, and PM equally contributed to the writing of the Editorial.

### Conflict of interest statement

The authors declare that the research was conducted in the absence of any commercial or financial relationships that could be construed as a potential conflict of interest.
